# Delayed Small Bowel Perforation in a Pre-Existing Ventral Hernia After Blunt Trauma

**DOI:** 10.7759/cureus.10150

**Published:** 2020-08-31

**Authors:** William D Tucker, Diane Cobble, Christy Lawson, Bracken Burns

**Affiliations:** 1 Surgery, East Tennessee State University Quillen College of Medicine, Johnson City, USA; 2 Trauma Critical Care/Surgery, East Tennessee State University, Johnson City, USA; 3 Surgery, East Tennesse State University Quillen College of Medicine, Johnson City, USA

**Keywords:** trauma, blunt abdominal trauma, hernia, small bowel injury

## Abstract

A hollow viscus injury is an uncommon but potentially dangerous intra-abdominal injury that can result from blunt abdominal trauma. It can be misdiagnosed in patient, particularly when the patient has other concerning findings. Also, diagnosis can be increasingly difficult in a patient with a pre-existing ventral hernia and chronic abdominal pain.

In this case we present a 66-year-old women, with a history of a large ventral hernia and chronic abdominal pain, who presented to the emergency department after a motor vehicle crash (MVC). Patient denied abdominal tenderness at the time of presentation and the initial computed tomography (CT) did not demonstrate any abnormal findings within the abdomen. Patient later began experiencing increased abdominal pain and presented with a small bowel perforation within the hernia that required a bowel resection and hernia repair.

A review of the literature reveals that not only are hollow viscus injuries rare but there appears to be few documented cases of viscus injuries occurring within a existing ventral hernia.

## Introduction

Blunt trauma accounts for a large percentage of the trauma activations at our level one state-designated center. Often, presentation of blunt abdominal trauma drives diagnostic focus toward liver and spleen lacerations because these are common injuries, but hollow viscus injuries are not entirely infrequent. Small bowel injuries occurred in 1.1% of all admissions after blunt trauma in a large multi-institutional study evaluating over 200,000 trauma admissions. Even more rare was the diagnosis of small bowel perforation, seen in only 0.3% of patients presenting after blunt trauma [1]. Imaging may demonstrate free air or free fluid in dependent portions of the abdomen; however, absence of radiographic findings does not exclude possibility of bowel injury. Hollow viscus injuries may present with signs of abdominal pain, voluntary guarding, and peritonitis. In exceptionally rare circumstances patients with pre-existing hernias will experience hollow viscus perforation within the hernia sac leading to diffuse abdominal pain, free air, free fluid, and often signs of sepsis. A review of the literature shows few cases of hollow viscus injuries occurring within existing inguinal hernias but no cases occurring within a ventral hernia. This case describes a patient with a pre-existing ventral hernia and chronic abdominal pain who developed a bowel perforation within the ventral hernia after a motor vehicle crash (MVC).

## Case presentation

A unrestrained 66-year-old Caucasian female presented from a rollover MVC after a prolonged extrication during the winter months. During the course of her extrication, she was partially submerged in a river. On initial evaluation, the patient was protecting her airway, had bilateral breath sounds, and palpable central pulses. Additionally, the patient presented with hypothermia, hypotension, and decreased mental status. Her temperature was 96.7 degrees Fahrenheit, her initial blood pressure was 74/45 and her Glasgow Coma Scale (GCS) was 9. Due to decreased mental status, a complete review of systems was unable to be completed. She did not have any abdominal tenderness at the time of secondary survey. Focused assessment with sonography for trauma (FAST) exam was negative. After IV fluid administration the patient's blood pressure improved to 152/71. At this point, computed tomographic (CT) imaging was obtained and showed a T12 compression fracture, facial fractures, and multiple right-sided rib fractures. After adequate resuscitation and rewarming the patient’s mental status improved and her GCS was 15. Her history was significant for a large chronic ventral hernia resulting from wound infection due to prior laparoscopic cholecystectomy. She also reported chronic abdominal pain and diarrhea related to this hernia over the last several years.

**Figure 1:**
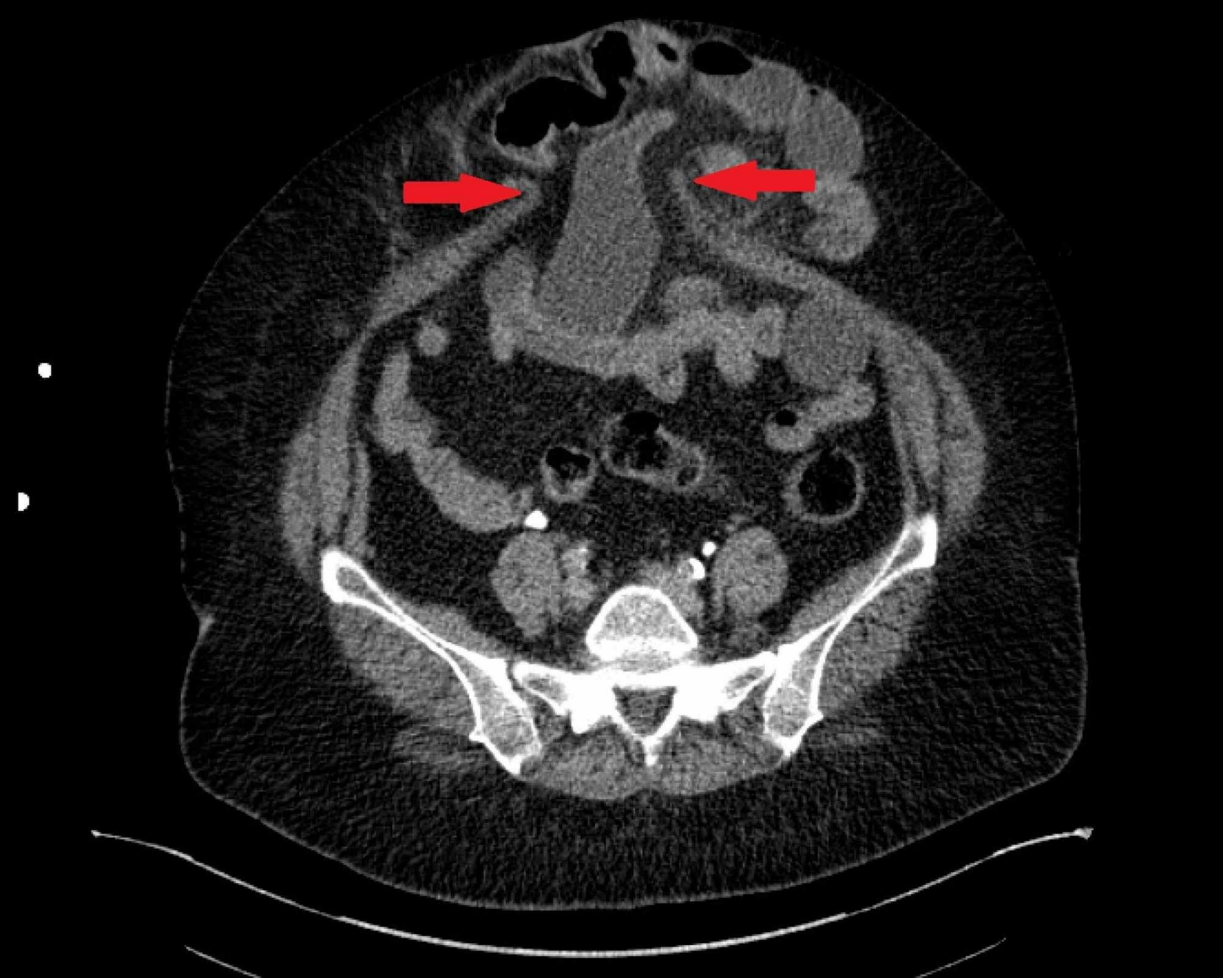
Initial CT scan demonstrating pre-existing ventral hernia (see arrows) with no free air, free fluid, intra-abdominal inflammatory changes, or other signs of perforated viscus

**Figure 2:**
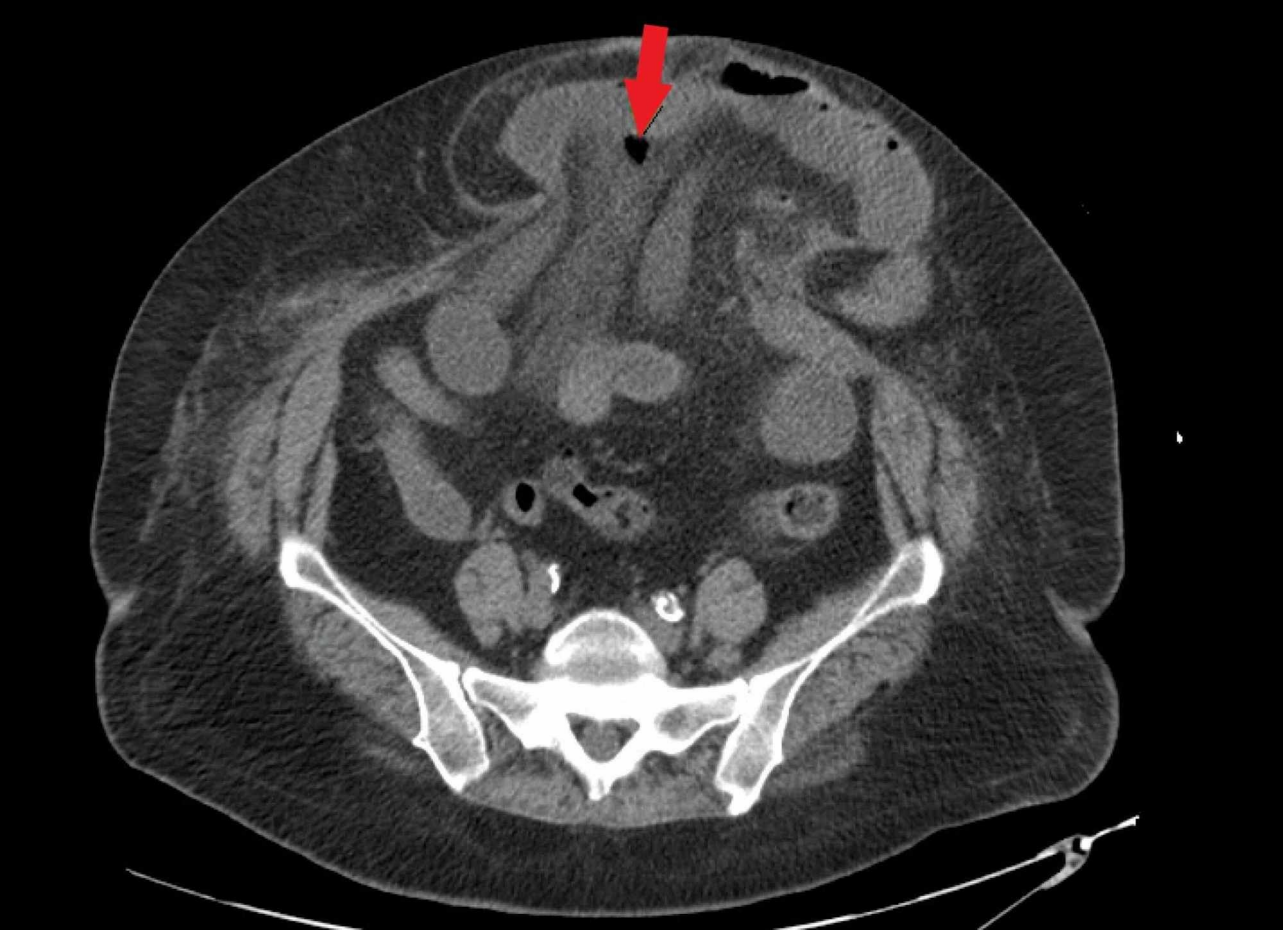
Repeat CT scan showing a small amount of free air (see arrow) adjacent to loops of small bowel within the ventral hernia and associated inflammatory changes

The initial CT scan did not demonstrate any free air, free fluid, intra-abdominal inflammatory changes, or other signs of perforated viscus (Figure 1). The patient's initial white blood cell count (WBC) was 24100/mm. It was also noted that the patient had a temperature of 101.8 degrees Fahrenheit during her first hospital night. Overnight she was mildly hypotensive with a systolic blood pressure around 85-105 mmHg and a mean arterial pressure of 55-65 which responded transiently to fluid resuscitation. The morning of hospital day two, the patient's WBC was 7900/mm. Physical exam on morning rounds the day after admission revealed the patient had become tender to palpation over her hernia and she reported mildly increased tenderness from her baseline. Eighteen hours after being seen on morning rounds the patient complained of worsening abdominal pain and a repeat CT scan was performed (approximately 27 hours after initial CT). This imaging now showed a small amount of free air adjacent to loops of small bowel within the ventral hernia as well as edema within the mesentery (Figure 2). The patient was taken to the operating room and a small bowel perforation was found within the ventral hernia along the sidewall of the bowel. The patient underwent a small bowel resection and primary ventral hernia repair with wound vac placement. The patient tolerated the procedure well. Her post-operative recovery was relatively uneventful. She was discharged to an inpatient rehabilitation facility and returned shortly after with abdominal pain, nausea, and vomiting. She was diagnosed with an ileus, had a short hospitalization with a return of bowel function and was discharged back to the rehabilitation facility.

## Discussion

Abdominal wall hernias are more commonly inguinal (75%) than ventral (10%) [2,3]. Strangulation and incarceration tend to be the most common serious complications of hernias. Some cases of traumatic abdominal hernia bowel perforations do exist in the current literature [3]. There are a few cases reporting ventral hernias complicated by delayed perforation after blunt trauma but none that we have found involve small bowel perforation within the hernia after a CT scan without evidence of small bowel injury [4]. The literature demonstrates that the presence of a hernia can be a risk factor for bowel perforation [5]. One case study presented a 55-year-old man who suffered abdominal trauma playing soccer. A CT scan revealed an existing inguinal hernia and free intra-peritoneal fluid. The patient was experiencing intense abdominal pain, hypotension and fever. This patient required emergency surgery which revealed ileum perforation with peritonitis. The intestinal perforation was repaired. The patient developed severe septic shock but recovered after three weeks [4]. Another case study presented a 54-year-old male who had experienced a minimal hit directly to his abdominal hernia. Patient presented with peritonitis and possible sepsis. An exploratory laporatomy was done and it was determined that patient had a bowel perforation. An intestinal resection and anastomosis was performed and recovery was uneventful. The hernia was repaired at a later operation due to peritonitis [6]. A study done by Vyas et al. reported that bowel perforation after blunt trauma was more likely to occur in patients over 40 years of age with a pre-existing hernia in comparison with healthy subjects of the same age [7]. Two theories exist to explain the underlying mechanism of perforation in these cases. One argues an increase in the intra-abdominal pressure and transfer of that pressure to the small bowel in a compressed space results in perforation [4,6]. The other theory argues shearing forces, when applied to abdominal hernias, particularly incarcerated hernias, result in direct injury to the hernia sac contents. A chronic low-grade inflammatory state found within an incarcerated hernia may prove to be a significant, but difficult to quantify, risk factor for perforation with blunt abdominal trauma [4]. 

Examination of all patients with significant blunt abdominal trauma at our institution consists of physical examination and a FAST exam. This test is well known to have certain strengths, like high specificity, and certain weaknesses, including low sensitivity and extreme variability with interuser operability. 

Computed tomography is often a test of choice for evaluation of the stable patient with blunt abdominal trauma [8]. Of all patients found to have perforated small bowel injuries, 13% had no findings indicative of small bowel injury on CT imaging [1]. Although imaging techniques and technology continue to improve, a negative CT scan cannot be relied on to conclusively eliminate a small bowel injury or a small bowel perforation. 

Small bowel perforation is a surgical emergency and requires prompt intervention. A delay in intervention greater than 24 hours has been reported to have a nearly quadrupled mortality compared to patients who had intervention within 24 hours [1].

## Conclusions

Traumatic bowel perforation is a rare, but well-known complication of blunt trauma and can be exceptionally difficult to diagnose. However, traumatic bowel perforation within a previously existing hernia is even rarer. A physician must consider a delayed presentation of bowel perforation within a trauma patient developing symptoms of sepsis even if initial imaging is negative. While acknowledging that these injuries are both rare and very difficult to diagnose, it is prudent to have an increased index of suspicion for small bowel perforation in a blunt trauma patient. It is also important to avoid anchoring to information that may support a diagnosis more comfortable to a clinician, but can rapidly cause clinical deterioration and harm to your patient.
